# Supplementary material for: Nodal signaling regulates asymmetric cellular behaviors, driving clockwise rotation of the heart tube in zebrafish
Source: Commun Biol. 2022 Sep 21;5:996. doi: 10.1038/s42003-022-03826-7 (PMC9492702; doi:10.1038/s42003-022-03826-7)
Supplement: Supplementary file 2 — Supplementary Information [file 42003_2022_3826_MOESM2_ESM.pdf]

SUPPLEMENTARY INFORMATION

Nodal Signaling Regulates Asymmetric Cellular Behaviors, Driving Clockwise Rotation of the Heart Tube in Zebrafish

Hinako Kidokoro, Yukio Saijoh, Gary C. Schoenwolf

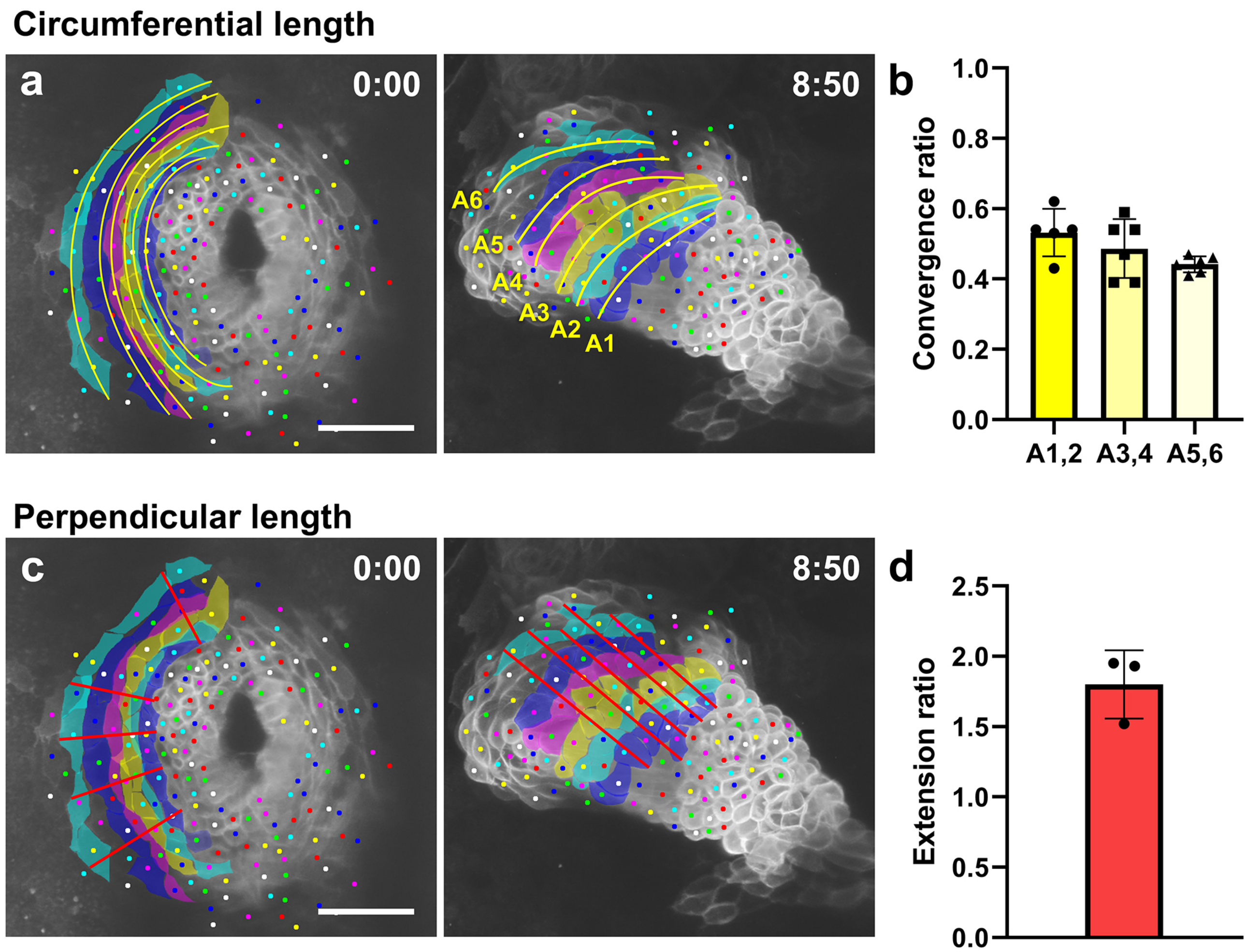

Supplementary Figure 1. The cardiac disc converges circumferentially while extending perpendicularly. (**a**) The circumferential length of each cell array (A1-A6, different colors for each cell array) was measured at the cardiac disc stage (0:00) and when the heart tube formed (8:50). The yellow lines indicate the measured circumferential lengths. Scale bar = 50 µm. (**b**) The convergence ratio was calculated by dividing the circumferential length of each array at 8:50 (h:min) by the comparable one at 0:00. Each cell array converged by approximately 50 % in 9 hours, with the arrays at the more peripheral positions converging slightly more than those at the more inner positions. 5-6 cell arrays were measured per one embryo (n = 3 embryos). Each dot represents the convergence ratio of each cell array. Error bars indicate s.d.. (**c**) The perpendicular length of the cardiac disc (0:00) and the heart tube (8:50) was measured. The measurements were performed at five positions (red lines) for each time point. The averages of the length at these five positions for each time point were calculated for each embryo (n = 3 embryos). These average values were used for calculating the extension ratio. Scale bar = 50 µm. (**d**) The extension ratio for each embryo was calculated by dividing the average of the perpendicular length of the heart tube at the 5 positions by that of the cardiac disc. Each dot represents the convergence ratio of each embryo. Error bars indicate s.d..

Fig. 4e

a. All (A1-A5)

b. Peripheral (A3-A5)

c. Inner (A1-A2)

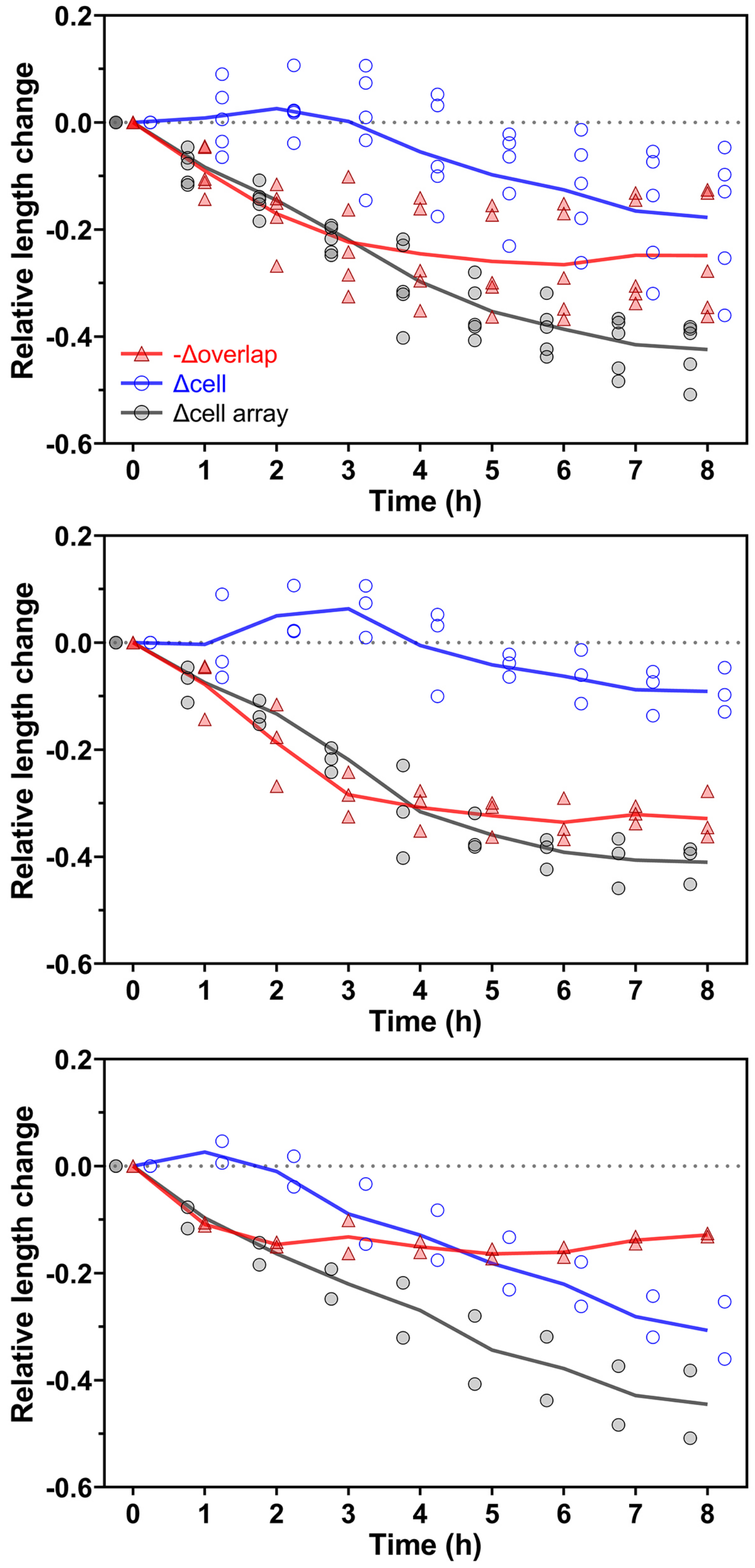

Supplementary Figure 2. Graphs corresponding to the line graphs shown in Figure 4e, showing individual data points with mean. (**a**) A graph corresponding to Figure 4e1. (**b**) A graph corresponding to Figure 4e2. (**c**) A graph corresponding to Figure 4e3. Each dot represents relative length changes of cell array (grey circle), cells (blue circle), and loss of the cell length by the cell overlap (red triangle) in each cell array of wild-type embryos. Solid lines indicate mean values for all (a), peripheral (b), and inner (c) cell arrays, respectively.

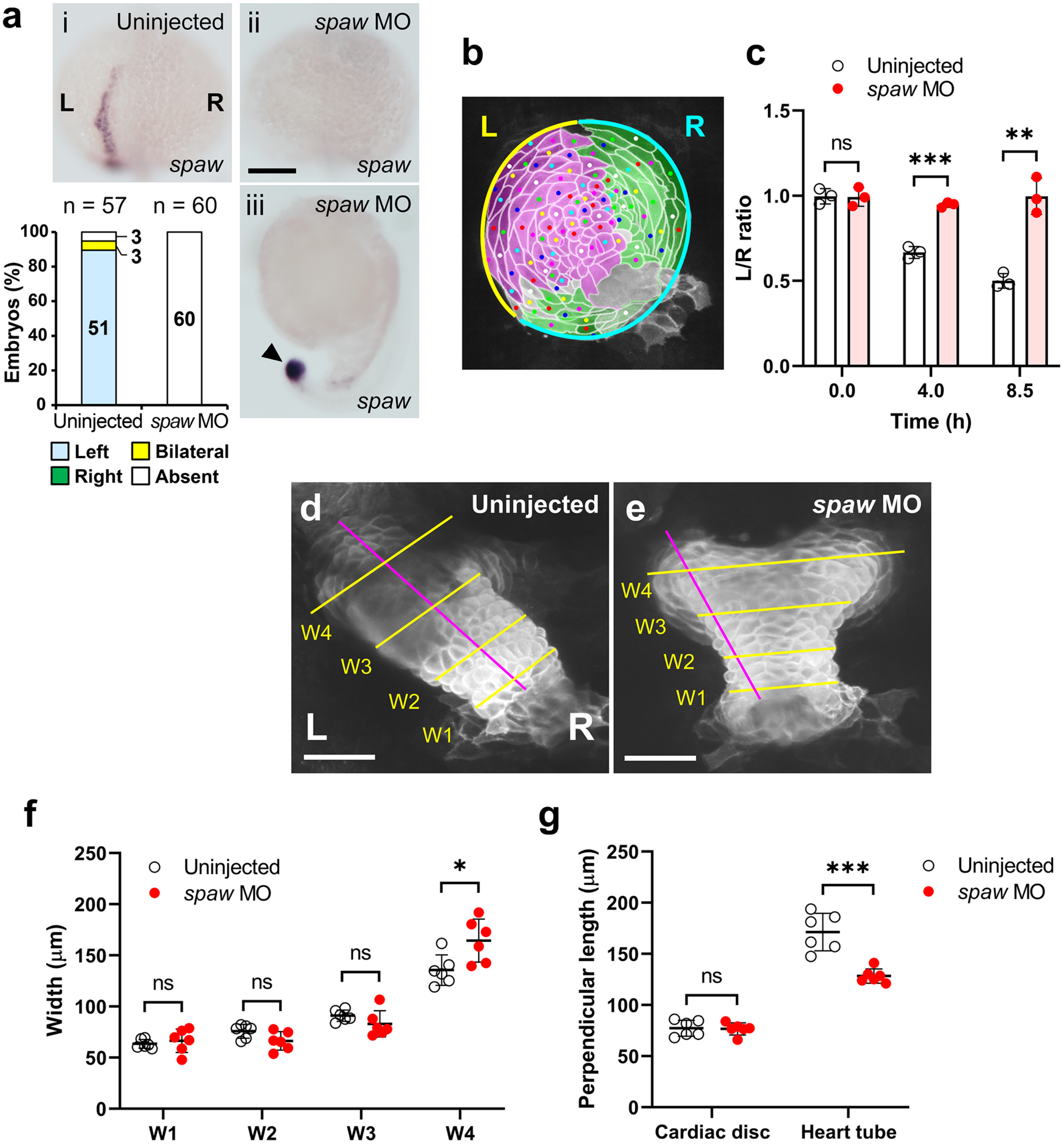

Supplementary Figure 3. Morphological comparison of the heart tube between uninjected and *spaw* MO-injected embryos. (**a**) Expression of *spaw* mRNA in uninjected siblings (i) and *spaw* MO-injected embryos (ii, iii). The bar graph shows percentage of control (uninjected siblings, n = 57), and MO-injected embryos (n = 60) with left-sided, right-sided, bilateral, or absence of *spaw* expression in the lateral plate mesoderm (LPM). Morpholino injection completely abolished *spaw* expression in the LPM (ii), whereas it remained normally expressed in the tail bud (iii, arrowhead). Scale bar = 200 µm. (**b, c**) The peripheral lengths (outline) of each left (yellow line in b) and right (cyan line in b) heart primordia were measured at the cardiac disc stage (0 h), during heart tube formation (4 h), and after the heart tube formed (8.5 h). The left (L)/right (R) ratio was calculated by dividing the peripheral length of the left primordium by that of the right primordium for each time point (c). The peripheral lengths of the left and right primordia were nearly equal at the cardiac disc stage, and the length on the left became significantly smaller than on the right as the heart tube formed in uninjected embryos (white dots in c, n = 3 embryos).

(*legend continued on next page*)

In contrast, the peripheral lengths of the left and right primordia in *spaw* MO-injected embryos (red dots in c, n = 3 embryos) were similar throughout heart tube formation. Each dot represents the L/R ratio of each embryo. Error bars indicate s.d.. The image shown in b is the same as the one shown in Figure 5a. (**d-g**) Quantification of the width (f) and length (g) of the heart tube in uninjected (d) and *spaw* MO-injected (e) embryos. The magenta line in d and e indicates the length of the heart tube. The widths of the heart tube were measured at four different positions (W1-4, yellow lines in d and e): the arterial end of the heart tube (W1), and the one-quarter (W2), one-half (W3), and three-quarters (W4) positions of the heart tube from the arterial side. These four positions were determined as follows: the heart length (magenta line in d, e) was divided into equal quarters. W2, W3 and W4 positions were set to one-quarter, one-half, and three quarters of the heart length, respectively. W1 position was set to the slightly (about one-cell row) venous (superior) side than the arterial (inferior) end of the magenta line, as the arterial end of the tube often was slightly folded outward. The heart tube widths at W1-3 positions were not significantly different between uninjected (n = 6, white dots in f) and *spaw*-MO injected (n = 6, red dots in f) embryos, whereas the widths at W4 position of *spaw* MO-injected embryos were significantly greater than that of uninjected embryos (f). The heart tube lengths of *spaw* MO-injected embryos (n = 6, red dots in g) were significantly smaller than that of uninjected embryos (n = 6, white dots in g), whereas the perpendicular lengths of the cardiac disc were nearly identical between uninjected and spaw MO-injected embryos. Each dot represents the heart width (f) and length (g) of each embryo. Error bars in f and g indicate s.d.. Scale bars in d and e = 50 μm. Unpaired two-tailed t-test with Welch's correction was used for statistical analysis in c, f, g. *$P<0.05$, **$P<0.01$, ***$P<0.005$. "ns" indicates not significant.

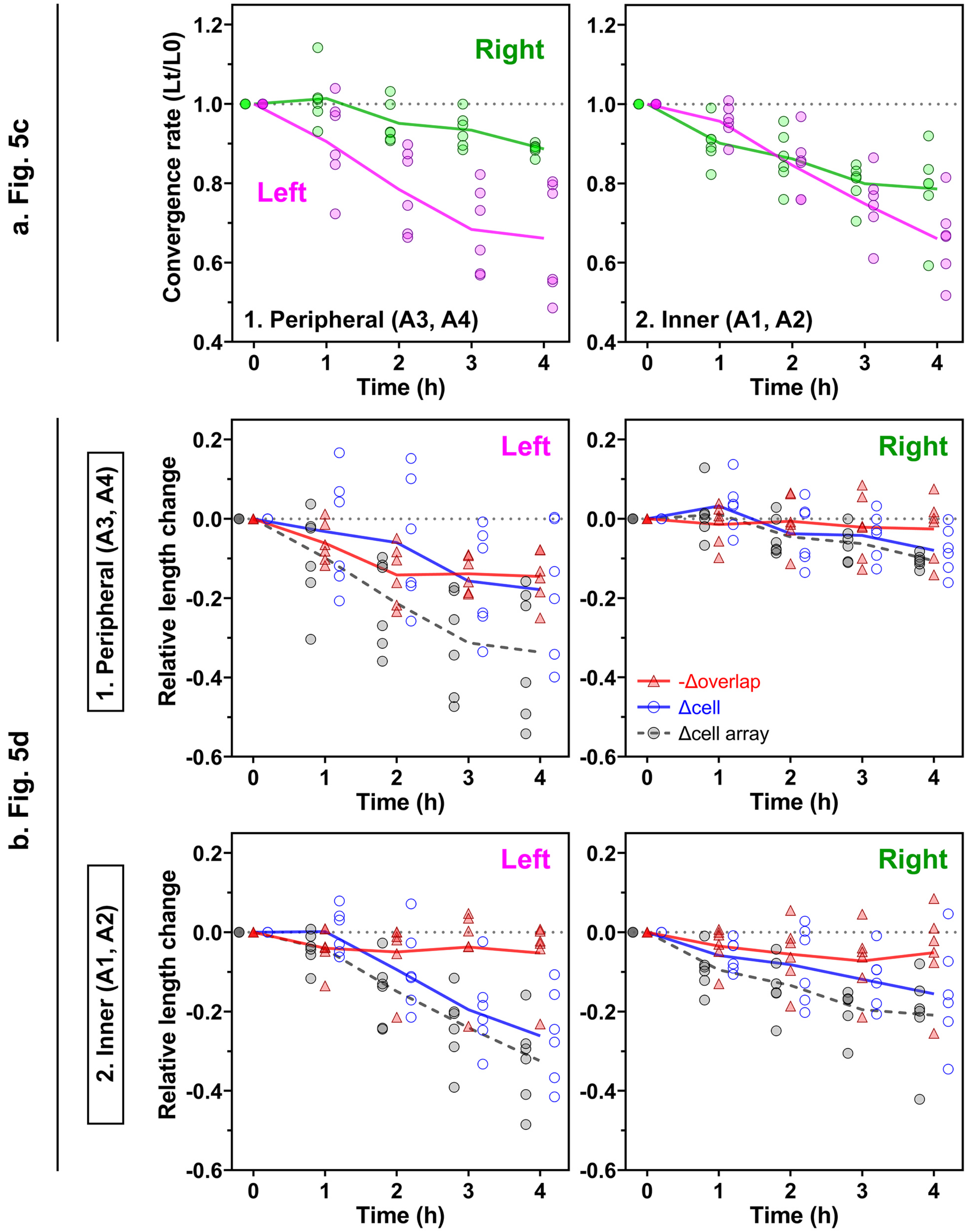

Supplementary Figure 4. Graphs corresponding to the line graphs shown in Figure 5c and 5d, showing individual data points with mean. (**a**) Graphs corresponding to Figure 5c. Each dot (magenta: left, green: right) represents the relative length (Lt/L0) of each cell array in wild-type embryos. Solid lines (magenta: left, green: right) indicate mean values for peripheral (1) and inner (2) cell arrays. (**b**) Graphs corresponding to Figure 5d. Each dot represents relative length changes of cell array (grey circle), cells (blue circle), and loss of the cell length by the cell overlap (red triangle) in each cell array of wild type embryos. Solid and dashed lines indicate mean values for peripheral (1), and inner (2) cell arrays.

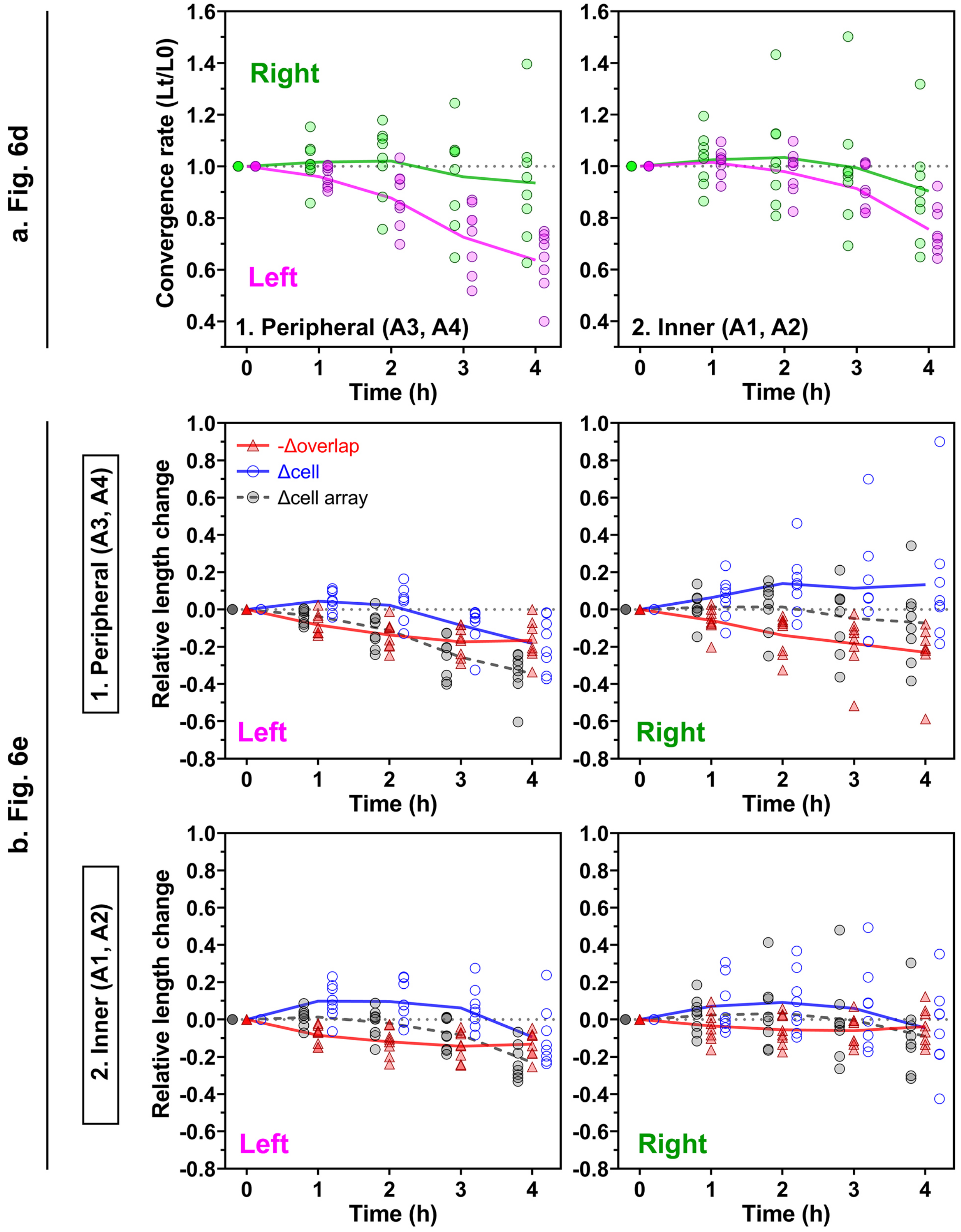

Supplementary Figure 5. Graphs corresponding to the line graphs shown in Figure 6d and 6e, showing individual data points with mean. (**a**) Graphs corresponding to Figure 6d. Each dot (magenta: left, green: right) represents the relative length (Lt/L0) of each cell array in embryos with cardia bifida. Solid lines (magenta: left, green: right) indicate mean values for peripheral (1) and inner (2) cell arrays. (**b**) Graphs corresponding to Figure 6e. Each dot represents relative length changes of cell array (grey circle), cells (blue circle), and loss of the cell length by the cell overlap (red triangle) in each cell array of embryos with cardia bifida. Solid and dashed lines indicate mean values for peripheral (1), and inner (2) cell arrays.

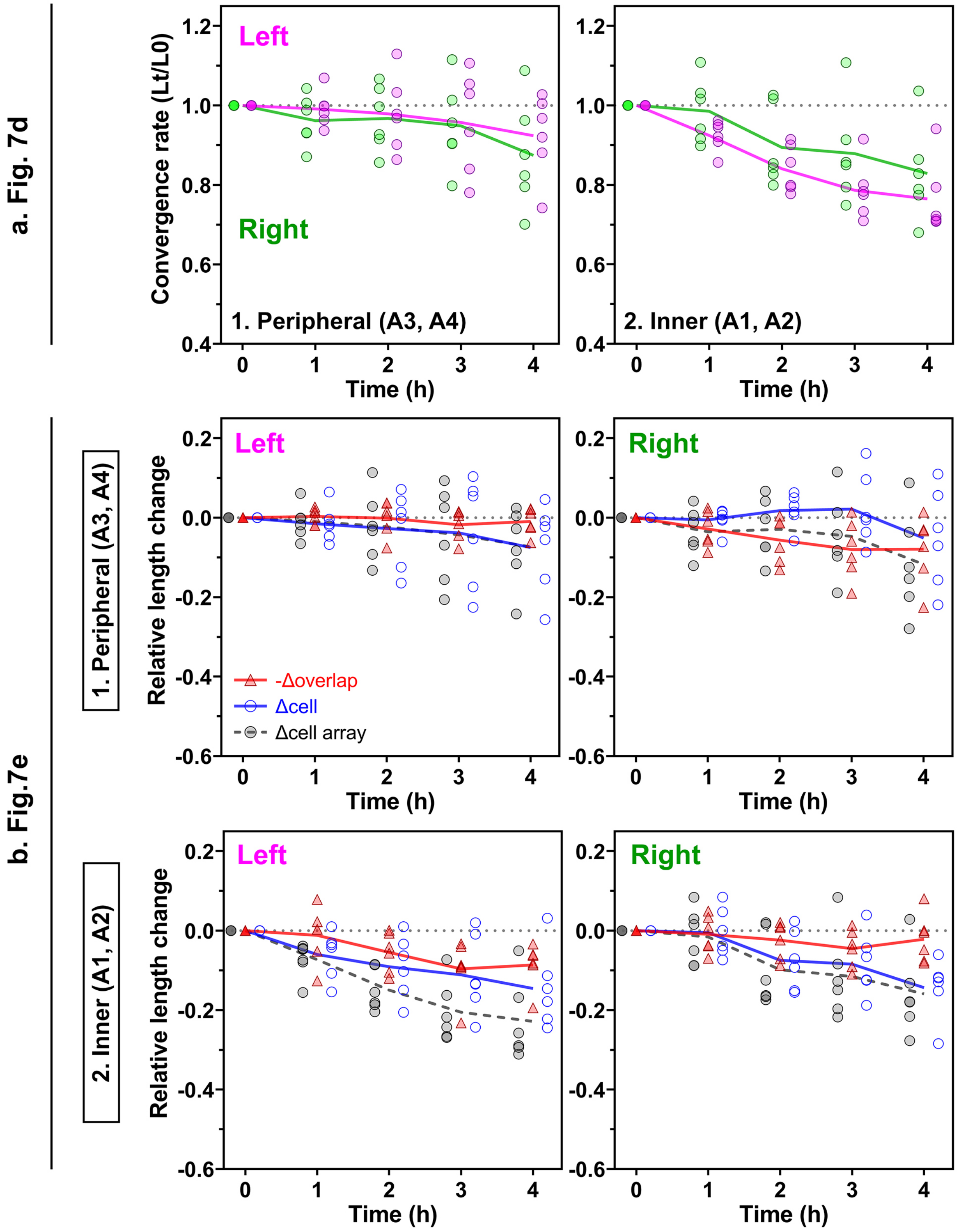

Supplementary Figure 6. Graphs corresponding to the line graphs shown in Figure 7d and 7e, showing individual data points with mean. (**a**) Graphs corresponding to Figure 7d. Each dot (magenta: left, green: right) represents the relative length (Lt/L0) of each cell array in *spaw*-MO injected embryos. Solid lines (magenta: left, green: right) indicate mean values for peripheral (1) and inner (2) cell arrays. (**b**) Graphs corresponding to Figure 7e. Each dot represents relative length changes of cell array (grey circle), cells (blue circle), and loss of the cell length by the cell overlap (red triangle) in each cell array of *spaw*-MO injected embryos. Solid and dashed lines indicate mean values for peripheral (1), and inner (2) cell arrays.

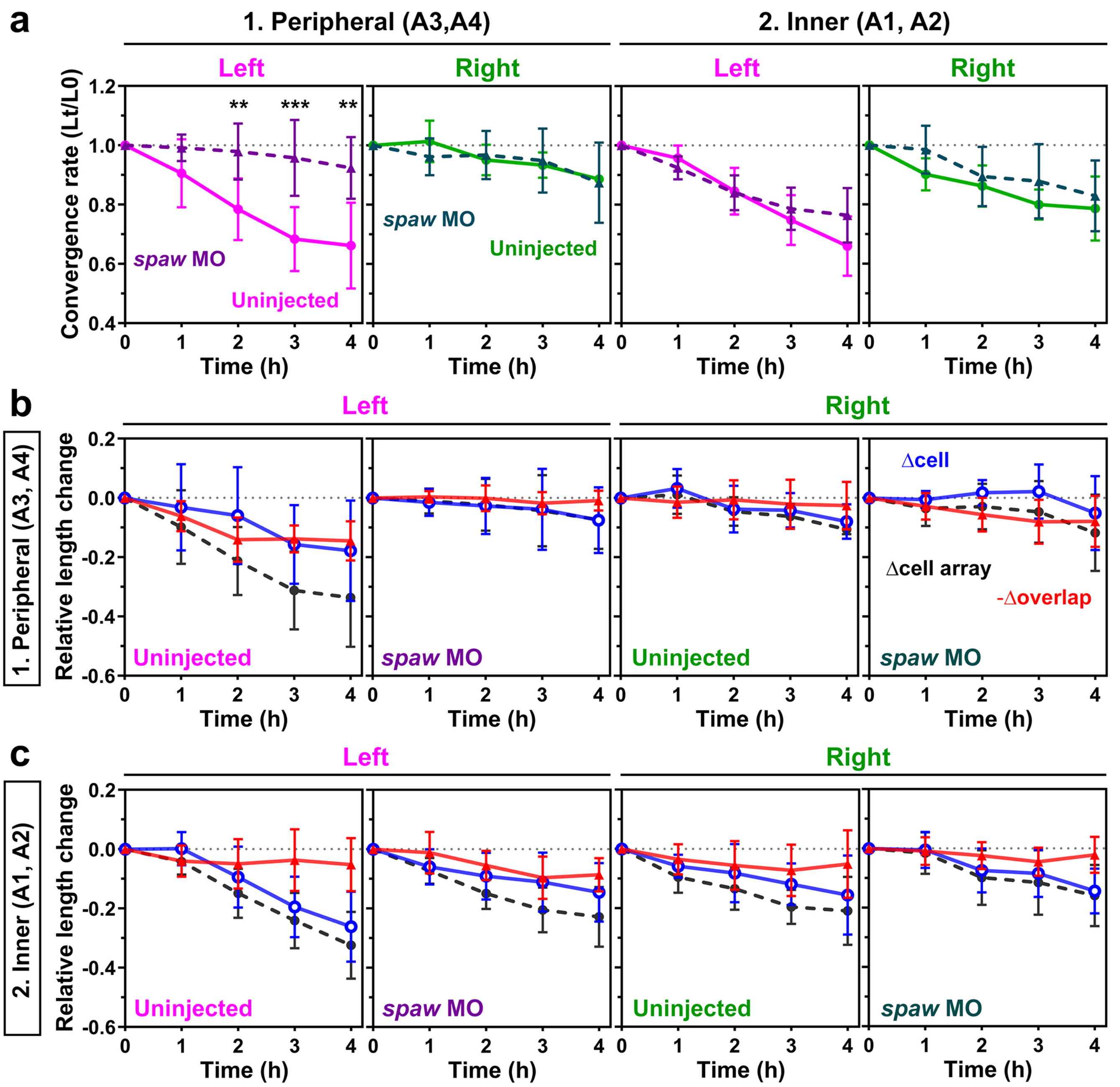

Supplementary Figure 7. Comparison of the circumferential convergence of the heart primordia between uninjected and *spaw* MO-injected embryos. The quantification data shown in Figures 5c, d (uninjected embryos), and 7d, e (*spaw* morphants) were combined and rearranged to compare the heart convergence between in the presence and absence of *spaw.* (**a**) Plots of the relative lengths (Lt/L0) of peripheral (1) and inner (2) cell arrays. The convergence of each left and right primordium was compared between uninjected and *spaw* MO-injected (*spaw* MO) embryos. Significant differences in the convergence rate were found in the peripheral region of the left primordium (a1, n = 3 embryos for each uninjected and *spaw* MO-injected embryos), whereas there were no significant differences in the inner region of the left primordium (a2) nor in the entire right primordium (a1 and a2). Two-tailed t-test assuming unequal variances was used for statistical analysis.*P<0.05, **P<0.01, ***P<0.005. Means±s.d. are shown. (**b-c**) Relative length changes of cell arrays (black), cells (blue), and loss of the cell lengths caused by the cell overlap (red) in the peripheral (b) and inner (c) cell arrays of each left and right primordium. Means±s.d. are shown.

Fig. S7a

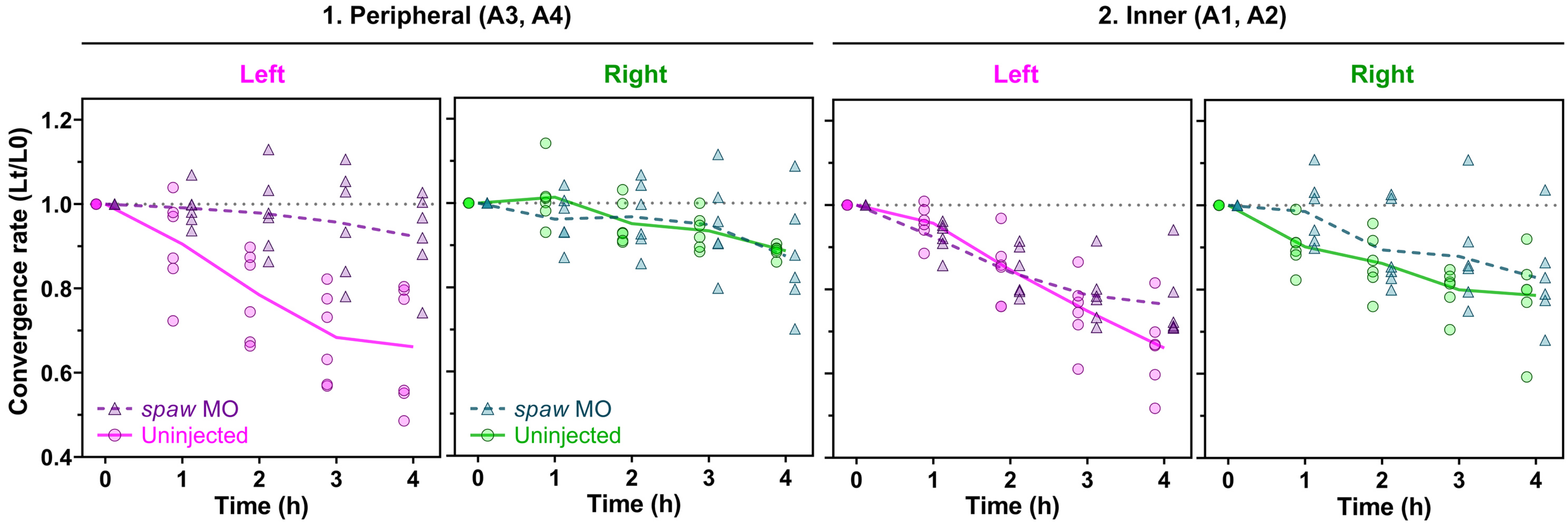

Supplementary Figure 8. Graphs corresponding to the line graphs shown in Supplementary Figure 7a, showing individual data points with mean. Comparison of the cardiac convergence between uninjected and *spaw* MO-injected embryos. Each dot (circle: uninjected embryos; triangle: *spaw* MO-injected embryos) represents the relative length (Lt/L0) of each cell array. Solid (uninjected embryos) and dashed (*spaw* MO-injected embryos) lines indicate mean values for peripheral (1) and inner (2) cell arrays.

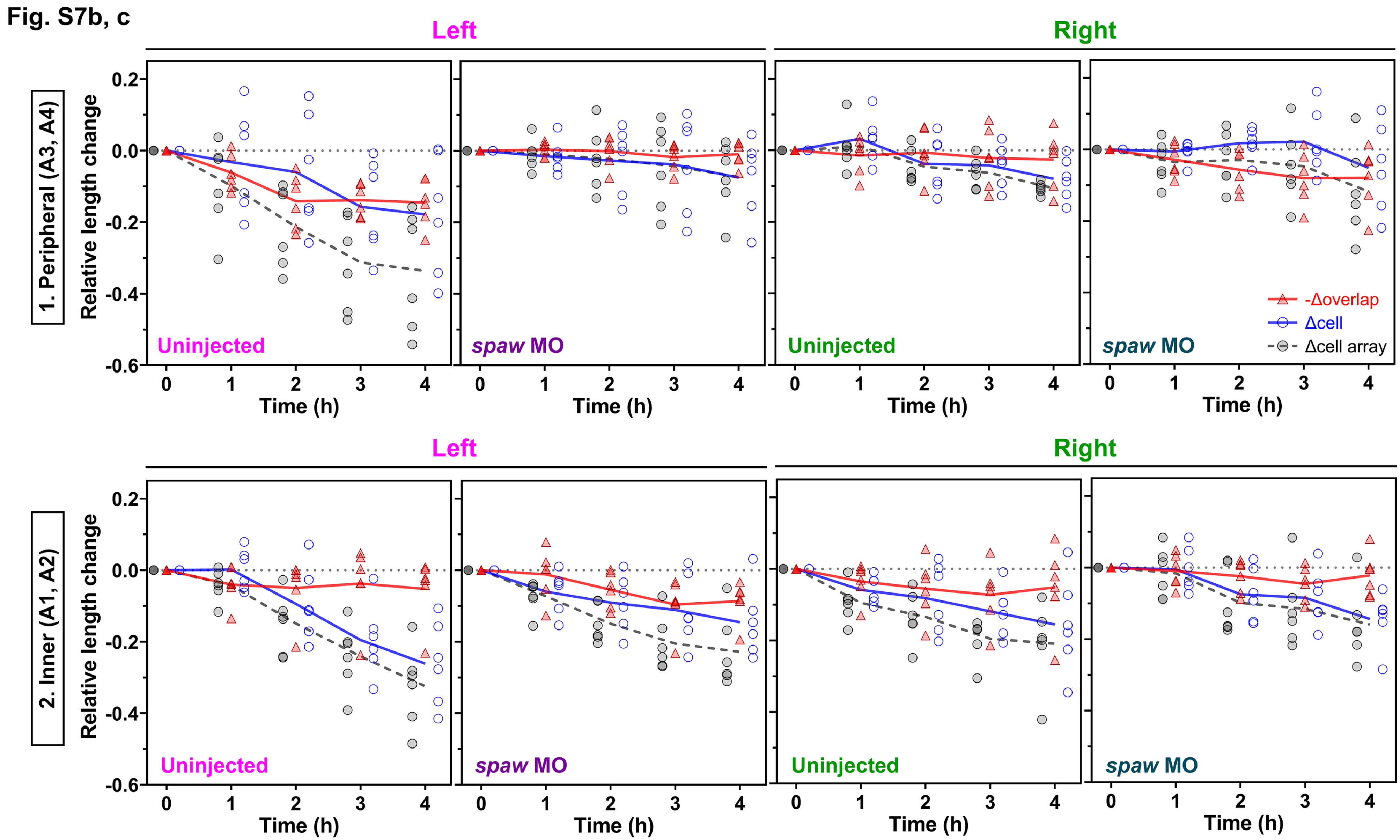

Supplementary Figure 9. Graphs corresponding to the line graphs shown in Supplementary Figure 7b and 7c, showing individual data points with mean. Comparison between uninjected and *spaw* MO-injected embryos. Each dot represents relative length changes of cell array (grey circle), cells (blue circle), and loss of the cell length by the cell overlap (red triangle) in each cell array. Solid and dashed lines indicate mean values for peripheral (1), and inner (2) cell arrays.

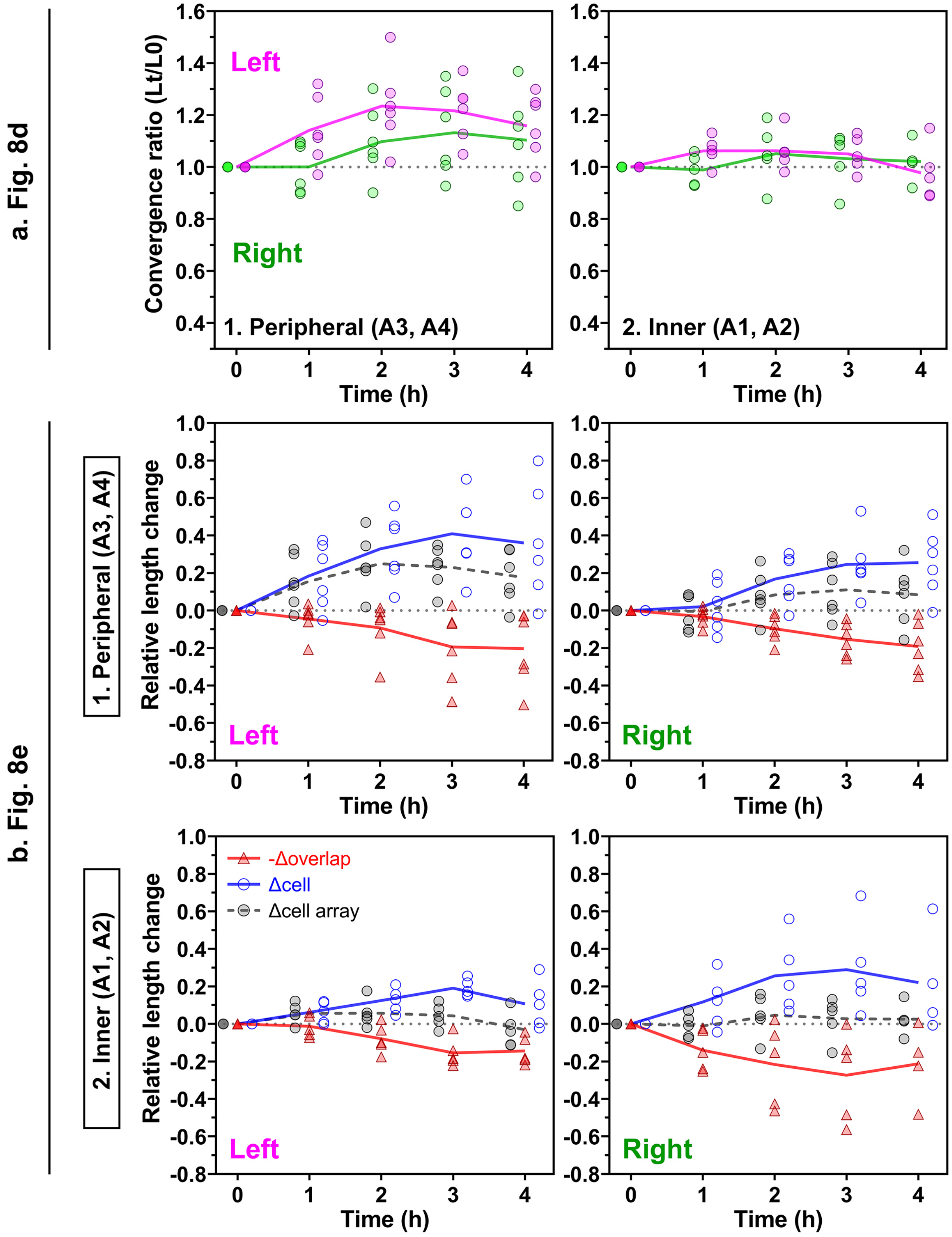

Supplementary Figure 10. Graphs corresponding to the line graphs shown in Figure 8d and 8e, showing individual data points with mean. (**a**) Graphs corresponding to Figure 8d. Each dot (magenta: left, green: right) represents the relative length (Lt/L0) of each cell array in *spaw* MO and *s1pr2* MO co-injected embryos. Solid lines (magenta: left, green: right) indicate mean values for peripheral (1) and inner (2) cell arrays. (**b**) Graphs corresponding to Figure 8e. Each dot represents relative length changes of cell array (grey circle), cells (blue circle), and loss of the cell length by the cell overlap (red triangle) in each cell array of embryos co-injected with *spaw* MO and *s1pr2* MO. Solid and dashed lines indicate mean values for peripheral (1), and inner (2) cell arrays.

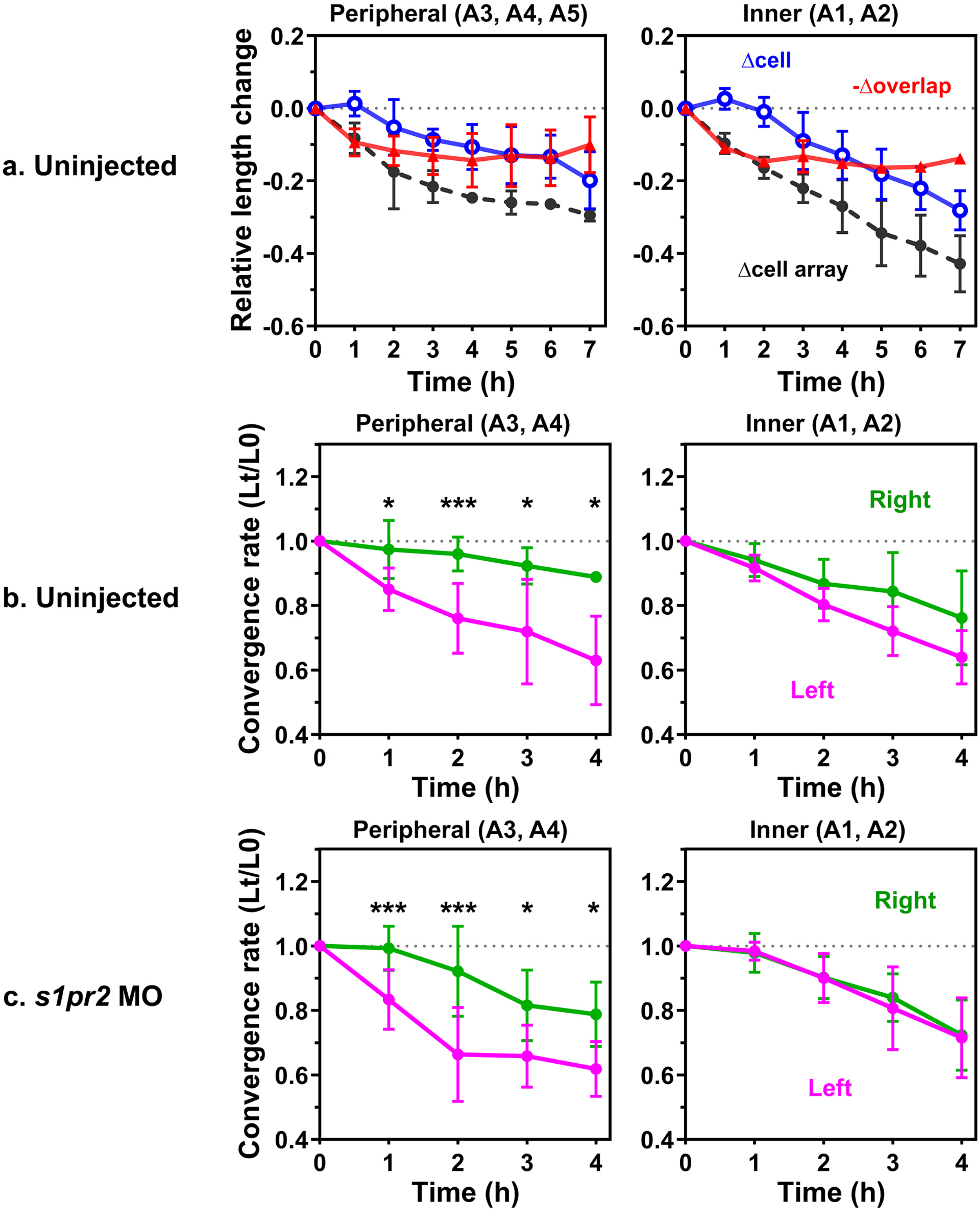

Supplementary Figure 11. The realignment of the measurements of Figures 4e, 5c and 6d. The measurements of Fig.4e (**a**), Fig.5c (**b**), and Fig. 6d (**c**) were realigned based on the initiation of cell shortening. (**a**) The realigned results of analysis of cell behaviors (Fig. 4e2, e3) showed similar, but not identical patterns, between peripheral and inner cells: inner cells displayed more cell length reduction, implying different cell properties between these regions. Means±s.d. are shown. (**b-c**) The realignment of Fig. 5c (b) and 6d (c) still showed significant differences in the convergence of cell arrays only in the peripheral regions, consistent with the original results. *P<0.05, **P<0.01, ***P<0.005 (two-tailed t-test assuming unequal variances). Means±s.d. are shown.

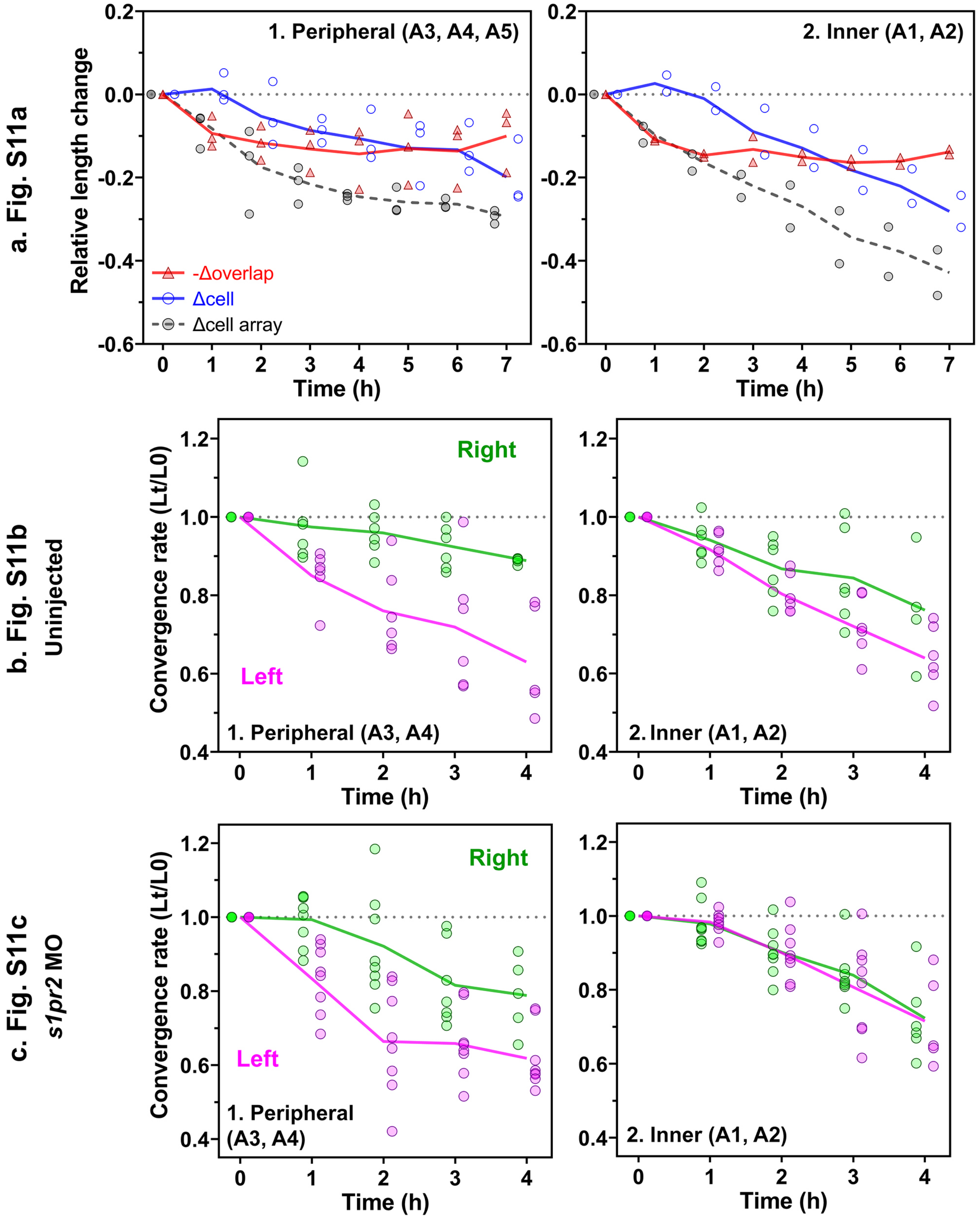

Supplementary Figure 12. Graphs corresponding to the line graphs shown in Supplementary Figure 11, showing individual data points with mean. (**a**) Graphs corresponding to Supplementary Figure 11a. Each dot represents relative length changes of cell array (grey circle), cells (blue circle), and loss of the cell length by the cell overlap (red triangle) in each cell array of wild-type embryos. Solid and dashed lines indicate mean values for peripheral (1), and inner (2) cell arrays, respectively. (**b**) Graphs corresponding to Supplementary Figure 11b. (**c**) Graphs corresponding to Supplementary Figure 11c. Each dot (magenta: left, green: right) represents the relative length (Lt/L0) of each cell array in normal embryos (b) and embryos with cardia bifida (c). Solid lines (magenta: left, green: right) indicate mean values for peripheral (1) and inner (2) cell arrays.
