# Supplementary material for: Nodal signaling regulates asymmetric cellular behaviors, driving clockwise rotation of the heart tube in zebrafish
Source: Commun Biol. 2022 Sep 21;5:996. doi: 10.1038/s42003-022-03826-7 (PMC9492702; doi:10.1038/s42003-022-03826-7)
Supplement: Supplementary file 3 — Description of Additional Supplementary Files [file 42003_2022_3826_MOESM3_ESM.pdf]

Description of Additional Supplementary Files

File name: Supplementary Data 1

Description: The source data underlying the graphs in the paper

File name: Supplementary Movie 1

Description: Confocal time-lapse imaging of a *Tg(myl7:EGFP-CAAX)^{ncv536Tg}* embryo, showing that the cardiac disc undergoes convergent extension during heart tube formation. Dorsal view (anterior to the top). Selected images are shown in Figure 2. Colored, duplicated time-lapse images are shown in the right panel. Relative times after the initiation of recording (h:min) are indicated in the upper right corner of each frame.

File name: Supplementary Movie 2

Description: Shape changes of the heart primordium during tube formation. Time-lapse video composed of only colored images from Supplementary Movie 1. Relative times after the initiation of recording (h:min) are indicated in the upper right corner of each frame.

File name: Supplementary Movie 3

Description: **Time-lapse tracking of myocardial cells in a Tg(myl7:EGFP-CAAX)^{ncv536Tg} embryo, showing intercalating myocardial cells (green) during heart tube formation**. Enlargement of colored cells are shown in the right panel. Selected images are shown in Figure 3a, b and c. Relative times after the initiation of recording (min) are indicated in the upper right corner of each frame.

File name: Supplementary Movie 4

Description: Cellular and tissue dynamics during convergence of the cardiac disc. The right panel shows the length change of a cell array. The left panel shows length changes of constituent cells and changes in

their arrangement. Selected images are shown in Figure 3d and 4c. Relative times after the initiation of recording (h:min) are indicated in the upper right corner of each frame.

File name: Supplementary Movie 5

Description: Asymmetric convergence of the left and right primordia during heart rotation. Cells derived from the left (magenta) and right (green) primordia are labeled with different colors. Only anterior parts of the primordia are colored. Related images are shown in Figure 5a. Relative times after the initiation of recording (h:min) are indicated in the upper right corner of each frame.

File name: Supplementary Movie 6

Description: Asymmetric convergence of the left and right primordia in cardia bifida. Confocal time-lapse recording of a *s1pr2* MO-injected *Tg(myl7:EGFP-CAAX)*^{ncv536Tg} embryo starting at 19-20 hpf. The two hearts still showed asymmetric convergence, independently of mechanical bias caused by heart rotation. Dorsal view (anterior to the top). Selected images are shown in Figure 6a. Relative times after the initiation of recording (h:min) are indicated in the upper left corner of each frame.

File name: Supplementary Movie 7

Description: Abolished asymmetric convergence of the left and right primordia in a *spaw* morphant. Confocal time-lapse recording of a *spaw* MO-injected *Tg(myl7:EGFP-CAAX)*^{ncv536Tg} embryo starting at 19-20 hpf. Spaw knockdown resulted in failure of the clockwise shift of the posterior border of the left and right primordia and heart rotation. In these morphants, the convergence of the left peripheral region of the heart primordium was significantly reduced as compared with that in uninjected embryos. Dorsal view (anterior to the top). Selected images are shown in Figure 7a.

Relative times after the initiation of recording (h:min) are indicated in the upper right corner of each frame.

File name: Supplementary Movie 8

Description: Abolished asymmetric convergence of the left and right primordia in a *spaw* morphant with cardia bifida. Confocal time-lapse recording of a *Tg(myl7:EGFP-CAAX)*^{ncv536Tg} embryo co-injected with *spaw* MO and *s1pr2* MO starting at 19-20 hpf. Loss of Nodal signaling abolished asymmetric convergence of the left and right heart primordia. Ventral view (anterior to the top). The images were

horizontally flipped to be presented in a consistent left-right orientation with other images and movies. Selected images are shown in Figure 8a. Relative times after the initiation of recording (h:min) are indicated in the upper right corner of each frame.
